# Patterns of alcohol and alcohol-flavoured non-alcoholic beverage advertisements over Japanese free-to-air television networks

**DOI:** 10.1186/s12889-022-14276-5

**Published:** 2022-10-06

**Authors:** Mio Kato, Hirono Ishikawa, Takahiro Kiuchi, Miki Akiyama, Yoko Kawamura, Tsuyoshi Okuhara, Naoko Ono, Rina Miyawaki

**Affiliations:** 1 Graduate School of Public Health, Teikyo University, 2-11-1 Kaga, Itabashi-ku, Tokyo, Japan; 2 Department of Health Communication, School of Public Health, The University of Tokyo, 7-3-1 Hongo, Bunkyo-ku, Tokyo, Japan; 3 Faculty of Environment and Information Studies, Keio University, 5322 Endo, Fujisawa-shi, Kanagawa Japan; 4 School of Health Sciences, University of Occupational and Environmental Health, 1-1, Iseigaoka, Yahatanishi-ku Kitakyushu-shi, Fukuoka, Japan; 5 Graduate School of Medicine, Juntendo University, 2-1-1, Hongo, Bunkyo-ku, Tokyo, Japan; 6 School of Arts and Letters, Meiji University, 1-9-1 Eifuku, Suginami-ku, Tokyo, Japan

**Keywords:** Alcoholic beverage, Alcohol-flavoured non-alcoholic beverages, SAFER, TV advertising, Adolescents, Marketing, Alcohol-free, Low-alcohol beverage

## Abstract

**Background:**

Alcohol use is a serious public health challenge worldwide. Japan has no government regulations or legal penalties against advertising alcoholic beverages on television (TV). Instead, advertisements depend on the Japanese alcohol industry’s self-regulation on airtime (no advertisements from 5 am to 6 pm) and the content of alcoholic beverages, which must not tempt minors. However, many adolescents (10 to 19 years old) watch TV from 6 pm to 11 pm. The aim of this study was to describe the pattern in the advertising of alcoholic beverages and alcohol-flavoured non-alcoholic beverages (AFNAB) in Japan during the popular TV viewing time for adolescents.

**Methods:**

A secondary analysis of advertising airtime data from five free-to-air Japanese TV networks in the Greater Tokyo area that aired between 12 August and 3 November 2019, was performed.

**Results:**

During the study period, 5215 advertisements for alcoholic beverages and AFNABs aired (1451.75 min). In total, 2303 advertisements (44.2%) were beer, low-malt beer, or beer-taste beverages, 277 (5.3%) were whisky, 2334 (44.8%) were local alcoholic beverages (*shochu* and *seishu*), and 301 (5.8%) were AFNAB. On average, more advertisements aired on weekends (67.6 advertisements) than on weekdays (59.3 advertisements) per day. Approximately 30% of advertisements for AFNABs were aired during the time restricted for alcohol advertising, although AFNABs are considered alcohol according to industry guidelines. During the popular television viewing time for young adolescents, about two to three times more advertisements were aired per hour than during the rest of the day, on both weekdays and weekends (*p* < 0.001).

**Conclusion:**

The number of alcohol advertisements aired at times when adolescents often watch TV is 2 to 3.2 times higher than that at other times of the day. Furthermore, despite the industry’s self-imposed regulations, some alcoholic beverages are still advertised. Therefore, other methods to protect children and adolescents from exposure to advertisements for alcoholic beverages should be investigated and implemented.

## Introduction

Alcohol use is a serious public health concern worldwide. Teenage drinking has been shown to give rise to negative effects not only at the time of drinking, but throughout life [1–3]. There are alcohol-related problems, such as alcohol-related harassment, unsafe sex, violence, non-communicable diseases, and job loss [4–7]. Therefore, the global community is strongly committed to reducing alcohol abuse at every stage of life [8]. One recommended strategy is to participate in the World Health Organization’s SAFER initiative, which includes five cost-effective interventions to reduce alcohol-related harm: (1)strengthening restrictions on alcohol availability, (2)advancing and enforcing drinking-and-driving countermeasures, (3)facilitating access to screening, brief interventions, and treatment, (4)enforcing bans or comprehensive restrictions on alcohol advertising, sponsorship, and promotion, and (5)raising prices on alcohol through excise taxes and pricing policies [9, 10].

Intervention number four listed above mentions prohibition on advertising for the sale of alcoholic beverages. An example of an intervention that enforces bans or implements comprehensive restrictions on alcohol advertising, sponsorship, and promotion is the regulation of advertising for alcoholic beverage marketing. Advertisements for alcoholic beverages are attractive not only to adults but also to minors, particularly teenagers, and increase their willingness to buy and consume alcohol [11–13]. Even short-term exposure of adolescents to alcohol advertisements is associated with positive thoughts about alcohol use and an increase in alcohol consumption [1–3, 14]. Such results have supported governments, civil societies, and other communities in many countries in their attempts to achieve full or partial bans on TV advertising.

In fact, approximately 80% of the G20 countries have legally binding regulations on alcoholic beverage advertising in any form of media, banning alcohol advertising in whole or in part [8].

The minimum legal age for purchasing and consuming alcoholic beverages in Japan is 20 years. However, Japan has no legally binding regulations for alcoholic beverage advertising in any form of media [15, 16]. Rather, alcohol advertising in the country is self-regulated by the Japanese alcohol industry through the voluntary standards of advertising, promotion of alcoholic beverages, and labelling of alcoholic beverage containers’ [17] and is exclusive. The Health and Medicine of the Alcohol Association’s Alcohol Beverage Advertising Judging Committee was formed by nine alcoholic beverage industry organisations: the Japan Sake and Shochu Makers Association, Nippon Distillers Association, Brewers Association of Japan, Japan Spirits & Liqueurs Makers Association, Japan Wineries Association, Japan Wholesalers Association, All Japan Liquor Merchants Association, Japan Wines and Spirits Importers’ Association, and Japan Brewers Association. The committee reviewed and approved any complaints regarding the advertising of alcoholic beverages and discussed compliance with standards and other issues.

According to Japanese industrial guidelines, TV advertisements for alcoholic beverages should not be aired from 5 am to 6 pm; therefore, advertisements for alcoholic beverages are allowed to be aired from 6 pm to 5 am. The self-regulatory guidelines include statements that TV and radio sponsors should be careful in including (1) advertisements for alcoholic beverages in programmes that are demonstrably produced with the plan that at least 70% of the programme’s audience will be aged 20 years or older; (2) spot advertisements for alcoholic beverages should be avoided as much as possible immediately before and after TV and radio programmes aimed at individuals aged younger than 20 years, and (3) no advertisements should be placed in TV programmes, radio programmes, newspapers, magazines, the internet or brochures aimed at individuals aged younger than 20 years. Japanese adolescents’ popular TV viewing time reportedly ranges from 6 pm to 11 pm on both weekdays and weekends and from 6 am to 9 am on weekdays [18]. Therefore, adolescents may be exposed to alcohol advertisements on TV during non-restricted hours.

The awareness that alcohol has effects on visceral fat and liver led to an increase of consumption of alcohol-flavoured non-alcoholic beverages (AFNABs) [19]. According to a recent report on the market for alcohol-free and low-strength beverages, the sale of alcohol-free and low-strength drinks in pubs, bars, and restaurants grew by 48% in the 12 months preceding November 2019 [20]. In 2019, the non-alcoholic beverage market in Japan was estimated to be approximately 22.43 million boxes (102% year-on-year), with the market size expected to increase in 2020 to approximately 22.66 million boxes (101% year-on-year) [21]. One box contains 20 633-mL bottles.

AFNABs might not be the sole primer for the onset of alcohol use in adolescents, although alcohol and AFNAB use have been significantly associated, and alcohol consumers have substituted AFNABs for alcoholic beverages to meet their needs [22]. The contradiction between the actual marketing message by the industry to sell because of their business goals and the self-regulation guidelines may hinder a clear message to TV viewers, including adolescents and their parents. In the guidelines, AFNAB is considered an ‘alcoholic beverage’; however, there is no specification for the time restriction of advertisement hours.

Industry self-regulation alone is known to be not enough to protect adolescents from the marketing messages of alcoholic beverages aimed at motivating them to drink [23–26]. Thus, this study examined the pattern of TV alcoholic beverage advertising practiced under self-regulation by industry associations, despite an exception from SAFER requirement No 4 to enforce prohibitions or broad restrictions on alcohol advertising, sponsorship, and promotion.

Therefore, our research questions (RQ) were:

RQ1: Are the self-regulated restrictions followed by the alcohol industry in advertising activities?

RQ2: Does alcohol advertising attempt to reach adolescents by increasing the number of advertisements when adolescents typically watch TV?

RQ3: Does the alcohol industry use sporting events to advertise alcoholic beverages more intensely?

RQ4: Does the alcohol industry use children/adolescent-oriented TV programmes to advertise alcoholic beverages?

## Methods

### Data of TV advertisements for alcoholic beverages and AFNABs

A database for November 2019 of TV advertisements was provided by an advertising monitoring company (Video Research Ltd., Tokyo, Japan). The database included advertisements of eight types of alcoholic beverages aired from 12 August to 3 November 2019 on five Japanese TV channels in the Kanto region of Japan: Nippon Television Network Corporation, TV Asahi Corporation, Tokyo Broadcasting System Television, TV Tokyo Corporation, and Fuji Television Network. There are more than 40 million residents of the Kanto region, constituting approximately 30% of the total Japanese population. In 2019, Japan hosted the Rugby World Cup for the first time; the dates of advertisements during this period included the six weeks of the big sports event ‘the Rugby World Cup’ (20 September to 2 November 2019) and six weeks before the event.

The database contains airdates, airtimes, duration of advertisements in seconds, manufacturers, product names, and types of beverages such as beer, low-malt beer, beer-taste beverages, wine, *seishu*, *shochu*, whisky, and non-alcoholic beer/spirit-flavoured beverages. *Seishu* and *Shochu* are the local beverages. *Seishu* is sake or Japanese rice wine. *Shochu* is a Japanese distilled beverage that contains less than 45% alcohol by volume. It is typically distilled from rice, barley, sweet potatoes, buckwheat, or brown sugar, and is categorised as a spirit elsewhere. Beer, low-malt beer, and beer-taste beverages were within one category in the database.

**Fig. 1:**
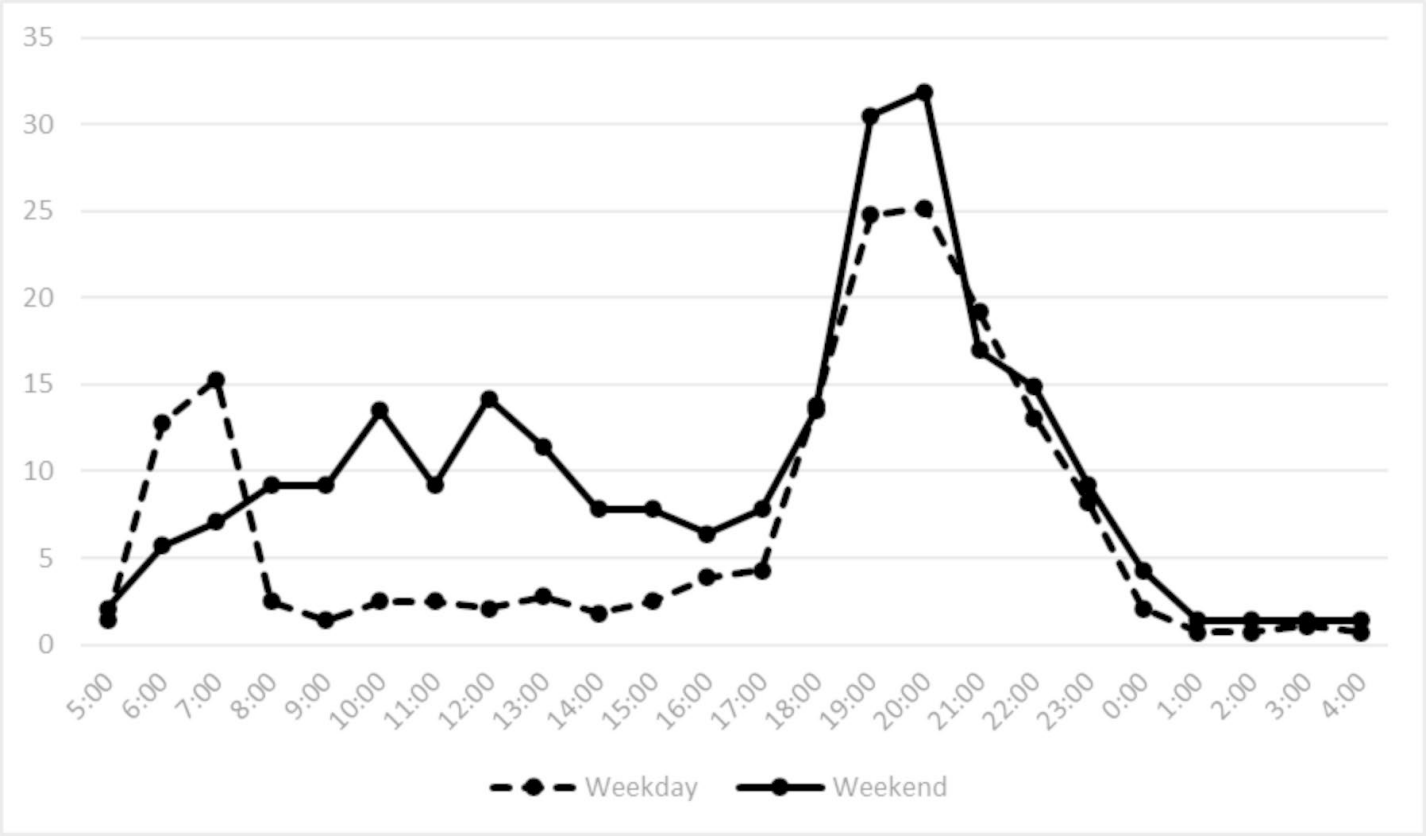
Percentages of adolescents (10 to 19 years old) of TV viewing on weekdays and weekends (created by the author from the data of Ministry of Internal Affairs and Information Communication)

The advertisements were coded according to whether they aired when the children and adolescents were likely to view them. In a study by Pettigrew et al., the TV viewing time popular with children was defined as the time of day when the proportion of children watching TV was 25% or more of the total expected child audience in Australia [12]. Available data published in Japan’s 2019 Survey Report on Information and Communication Media Usage Time and Information Behaviour relate to viewers aged 10 years and older. Following the Australian study, we defined adolescents’ popular TV viewing time (APVT) as the time when the proportion of adolescents (aged 10–19 years old) watching TV was more than 10% of the total possible adolescent audience. According to government statistics, APVTs range from 6 a.m. to 8 a.m. and from 6 p.m. to 11 p.m. on weekdays and from 6 p.m. to 11 p.m. on weekends (Fig. 1).

This study was exempted from institutional board review because human participants were not enrolled.

### Data analysis

The total number of advertisements were summed by type and channel during the study period of 84 days. The number of advertisements was also categorised into weekdays (56 days) and weekends (Saturdays, Sundays, and national holidays, which constituted 12, 12, and 4 days during the study period, respectively). To produce an average number of advertisements per day on weekdays and weekends, the number of advertisements was divided into 56 days for weekdays, 24 for Saturdays and Sundays, and 4 for holidays. Based on the studies by Pettigrew [12], the density was standardised as follows: As APVT constitutes a substantially shorter time period (approximately 7 h per day) than non-APVT (approximately 17 h per day), the density rates were standardised by dividing the number of advertisements by the number of hours in the viewing period and multiplying the resulting number by 24 to obtain a 24 h equivalent figure. In order to determine the density of alcohol and AFNAB advertisements placed in programmes recommended for adolescents during the hours when adolescents most frequently watch television, the number of alcohol and AFNAB advertisements aired within recommended programmes during APVT was divided by the number of alcohol and AFNAB advertisements aired during the entire APVT, and multiplied by 100 to yield a percentage. In addition, the same calculation was carried out for each TV station to show differences between stations.

Descriptive statistics were performed on the number of advertisements categorised by alcoholic beverages and AFNABs. The χ^2^ test was applied to compare the percentages of the number of advertisements within the alcohol and AFNAB categories as well as the percentages of the number of advertisements between the APVT and non-APVT periods. All analyses were conducted using the Statistical Package for the Social Sciences for Windows version 24 (SPSS IBM Corp., Armonk, NY, USA), and p-values less than 0.05 were considered as significant.

## Results

During the study period of 84 days, 5215 advertisements for alcoholic beverages and AFNABs were aired on five TV channels in the Kanto region (Greater Tokyo). Table 1 shows the distribution of advertisements based on the type of alcoholic beverage shown. Most alcohol advertisements featured local beverages (2334, 44.8%), followed by beer, low-malt beer, beer-taste beverages (2303, 44.2%), AFNABs (301, 5.8%), and whisky (277, 5.3%). No wine advertisements aired during the study period. There were 56 weekdays and 28 weekends during the study. Four national holidays (12 August, 16 September, 23 October and 14 October) were considered during the study period. Therefore, the advertisements per day on average were 59.3 on weekdays and 67.6 on weekends.

**Table 1:** Number of television advertisements by types of alcoholic beverage

	Total		Weekdays	Weekends	Weekday		Weekend	
	**(84 days)**		**(56 days)**	**(28 days)**	**(1 day)**		**(1 day)**	
	N	%	N	N	N	%	N	%
**Total**	5215	100.0	3321	1894	59.3	100.0	67.6	100.0
**Alcohol bev.**	4914	94.2	3148	1766	56.2	94.8	63.1	93.2
*Beer, low-malt beer, beer-taste bev.*	*2303*	*44.2*	*1343*	*960*	24.0	*40.4*	34.3	*50.7*
*Whisky*	*277*	*5.3*	*158*	*119*	2.8	*4.8*	4.3	*6.3*
*Local bev. (shochu, seishu)*	*2334*	*44.8*	*1647*	*687*	29.4	*49.6*	24.5	*36.3*
**AFNAB**	301	5.8	173	128	3.1	5.2	4.6	6.8

bev., Beverage. AFNAB, alcohol-flavoured non-alcoholic beverage. No wine or other alcoholic beverages were advertised

On weekdays, most advertisements featured local beverages, such as *shochu* and *seishu* (29.4, 49.6%), followed by beer, low-malt beer, beer-taste beverages (24.0, 40.4%), AFNABs (3.1, 5.2%), and whisky (2.8, 4.8%). On weekends, most advertisements featured beer, low-malt beer, or beer-taste beverages (34.3, 50.7%), followed by local beverages (24.5, 36.3%), AFNABs (4.6, 6.8%), and whisky beverages (4.3, 6.3%). During the study period, 301 advertisements for AFNABs were aired, of which 83 (27.6%) were aired from 5 am to 6 pm, which were restricted times according to industry guidelines, and 218 (72.4%) were aired from 6 pm to 5 am (data not shown).

On an average weekday, 86.6 alcohol and AFNAB advertisements were aired during APVT and 48.1 during non-APVT hours, and on an average weekend, 149.0 alcohol and AFNAB advertisements were aired during APVT and 46.2 during non-APVT hours (Table 2). The density of alcohol advertisements alone during APVT was 2.6 times higher than that during non-APVT. The results showed that there were 1.8 times more advertisements on weekdays and 3.2 times more advertisements on weekends when the adolescents watched TV during the APVT than during the non-APVT.

**Table 2:** Density and distribution of television advertisements in APVT and non-APVT

	Weekday			Weekend		
	Standardisesd APVT	Standardised Non-APVT	APVT/Non-APVT ratio	Standardisesd APVT	Standardised Non-APVT	APVT/Non-APVT ratio
Total	**86.6**	**48.1**	**1.80**	**149.0**	**46.2**	**3.23**
Alcohol	**82.6**	**45.4**	**1.82**	**141.8**	**42.4**	**3.34**
*Beer, low-malt beer, beer-taste beverage*	33.3	20.1	**1.66**	77.7	22.9	**3.39**
*Whisky*	5.1	1.9	**2.68**	12.3	2.1	**5.86**
*Local bev. (shochu, seishu)*	44.2	23.3	**1.90**	51.8	17.4	**2.98**
AFNAB	**4.0**	**2.7**	**1.48**	**7.2**	**3.9**	**1.85**

APVT Weekdays 6 am – 8 am, 6 pm – 11 pm (7 h), Weekends 6 pm – 11 pm (5 h)

Non-APVT Weekdays 17 h, Weekends 19 h

bev, beverage; AFNAB, alcohol-flavoured nonalcoholic beverage; APVT, adolescents’ popular viewing time

On weekdays, there were four advertisements of AFNABs during the standardised APVT and 2.7, during non-APVT, and on weekends, 7.2 during APVT and 3.9 during non-APVT. There were no significant differences between weekdays and weekends (*p* = 0.18), or between the APVT and non-APVT groups (*p* = 0.06).

During the Rugby World Cup in Japan, there were 17 matches broadcast on a private terrestrial TV, on which 13 alcoholic beverage advertisements were run. The number of advertisements on average per day was 53.4 before the event (12 August to 19 September, i.e., 39 days), whereas it was 68.1 during the event (20 September to 3 November, i.e., 46 days). Advertisements of whisky during the event were significantly more frequent than in the period before the event (4.6 ads vs. 1.7 ads, respectively, *p* = 0.045). Advertisements of AFNABs were significantly more during the event than in the period before the event (6.2 ads vs. 0.4 ads, respectively, *p* = 0.01). Local beverages also had more advertisements during the event than before the event (32.3 ads vs. 21.7 ads, respectively, *p* = 0.046). The density of advertising for beer, low-grade malt and beer-flavoured beverages alone decreased during the event compared to the level before it (29.5 ads vs. 25.0 ads, respectively, *p* = 0.002).

Each of the five TV stations in this study recommended five to seven programmes to promote the knowledge, understanding, and emotional enrichment of adolescents, resulting in a total of 30 programmes. Among these, seven programmes that aired between 5 am and 6 pm (prohibited hours by the industry self-regulation guidelines), and two animated programmes for children (cartoons) aired after 6 pm, did not advertise alcoholic beverages or AFNABs. However, in the remaining 21 programmes, 241 advertisements for beer, low-malt beer, beer-taste beverages, local beverages (*shochu* and *seishu*), whisky, and AFNABs (equivalent to 10.6% of the 2,284 advertisements for alcoholic beverages and AFNAB placed in APVT during the study period) were aired between the start and end of the programmes’ broadcast time. The density of alcohol and AFNAB advertisements varied among five stations, ranging from 1.0 to 8.4%. Thus, the results showed that advertising was not thoroughly regulated by the industry’s self-regulatory guidelines.

## Discussion

In response to our RQ1 regarding the self-regulation of advertising activities, alcohol industry TV advertising partially followed self-regulation restrictions. The Alcohol Association Alcohol Advertising Review Committee established Japanese Alcohol Industry Guidelines in December 1988. The Industry Self-Regulatory Guidelines were revised, most recently in July 2016, to keep up to date. Industry self-regulation has been said to be ineffective or inadequate in terms of minimising incentives to consume alcoholic beverages [14, 24, 25, 27]. The survey found that TV advertising of alcoholic beverages was in line with the industry’s voluntary standards, and confirmed that no alcoholic beverages were advertised on free-to-air TV during restricted advertising hours.

Conversely, although the voluntary standards concerning AFNABs clearly state that they are to be treated as alcoholic beverages, there is no specification of the advertising time, and almost 30% of the advertisements were aired within the prohibited advertising times for alcoholic beverages. This is worrisome because AFNAB advertisements can tempt adolescents to try alternatives to alcoholic beverages [22]. Therefore, the advertising of AFNABs should be restricted by current advertising time restrictions or banned as a further step.

Because the industry adheres to their self-regulation of advertisement times, advertising has become concentrated outside the restricted hours, which is what we asked in RQ2. A large amount of alcohol advertising was observed outside the restricted hours (from 6 pm onwards); however, from 6 pm onwards, families typically gathered, for example, to have dinner while watching TV. According to the Ministry of Health, Labour, and Welfare Science Study on Smoking and Drinking Status of Minors [28], the most common triggers for drinking among middle and high school students (aged 12–18 years) were weddings and funerals (25.7%), followed by those with family members (21.0%). In both cases, it is suspected that adults (parents or relatives) encourage minors to drink, which may be because of a lack of adult awareness. In addition, the number of advertisements was 3.5 times higher on weekends than weekdays. The most worrying aspect is that adolescents and even schoolchildren (6–12 years old) are exposed to alcoholic beverage advertisements as they watch TV until late at night.

In RQ3, we hypothesised that the alcohol industry uses major sporting events to advertise alcoholic beverages more intensely. Thirteen advertisements aired during the Rugby World Cup match on the TV. The reason for this low number could be that nine matches were broadcast from 5 am to 6 pm, in which Heineken (a beer manufactured and distributed by the Kirin Brewery in the Japanese market) is a worldwide sponsor, the Heineken Bar Finder Apps were promoted, and hawker salespeople with brand logos were stealthily visible to viewers during the games. ‘Hawker sales’ is a system whereby hundreds of sales staff carry drink containers with brand logos on their backs and walk around the venue, allowing customers to make purchases while they are in their seats watching the match. The system has been used in Japan for major professional sports matches to avoid queues at shops, but this was the first time it was used at the Rugby World Cup. All 13 terrestrial TV advertisements were in the beer, low-malt beer, and beer-taste beverage categories. Therefore, our findings do not directly implicate any additional influence of global sports events on TV advertisements. However, we observed that indirect ways in which the companies use sporting events to promote alcoholic beverages.

RQ4 assessed whether the alcohol industry advertised alcoholic beverages on TV programmes aimed at children and adolescents. Alcohol advertisements have appeared on children- and adolescent-oriented TV programmes that promote knowledge, understanding, and emotional development. However, it is unclear whether the alcohol industry deliberately placed advertisements because the programmes had good ratings, whether the alcohol industry knew that the advertisements were being placed during restricted broadcast hours and did not inform the producers of the TV programmes, or whether neither industry was aware of this discrepancy. This suggests that the alcohol industry’s voluntary standards may not be effective. Although the current study was based on data from alcohol and AFNAB advertisements only, more conclusive indications could be made by comparing advertisements aired in future adolescent-oriented TV programmes with other advertisements aired during APVT.

This study had three main limitations. Firstly, a short time period was covered: 84 days (12 August to 3 November 2019) during summer and autumn in Japan. Cultural and social events in Japan in the spring, summer, and winter seasons increase opportunities for drinking. More advertisements may air during the year. Secondly, only advertisements aired in the Kanto region are included. The advertising pattern in the Kanto region may have differed from that in other regions. However, this region was chosen because it has the largest population in Japan and, therefore, reaches a wider audience. Thirdly, we did not analyse TV advertisements on any particular TV programme that targeted a specific audience in terms of age or gender. TV programmes are produced for a specific audience and advertisements are introduced to target the audience. According to the Voluntary Advertising Standards, breweries are only allowed to advertise on TV programmes in which adults are the main target audience. However, primary school children also watch quizzes, and the comedy is aired outside of the restricted advertising times. A certain number of alcohol and AFNAB advertisements were aired during programmes that TV stations recommended young children and adolescents to watch. Therefore, there is inconsistency between industry self-regulation statements and actions. Further research is needed on effectiveness of industry self-regulation in Japanese media environment.

## Conclusion

The alcohol industry follows self-regulatory guidelines and strictly adheres to restrictions on advertising time for alcoholic beverages. However, these guidelines do not protect the health of consumers, particularly that of adolescents. Firstly, the timeframe for advertising restrictions was problematic. Although the guidelines state that no advertisements for alcoholic beverages should be aired before 6 pm, for Japanese adolescents, the period between 6 pm and 11 pm is when they eat dinner with their families and watch TV; thus, they are exposed to many alcohol and AFNAB advertisements. Secondly, there is inconsistency in the subject matter of restrictions within this guideline. Although the guidelines state that AFNABs should be treated as alcoholic beverages, they do not unify the time restrictions for TV advertisements with those for alcohol. Finally, there was an inconsistency between the Voluntary Advertising Standards, wherein breweries were only allowed to advertise on TV programmes where adults were the main target audience; however, alcoholic beverage advertisements were aired during TV programmes recommended for adolescents. Therefore, stronger measures, such as a total ban on advertising, will need to be considered to protect children and adolescents from overexposure to alcoholic beverages and AFNAB advertising.

## Data Availability

The data that support the findings of this study are available from Video Research Ltd., but restrictions apply to the availability of these data, which were used under licence for the current study and thus are not publicly available. However, data are available from the authors upon reasonable request with permission from Video Research Ltd.

## List of abbreviations

AFNAB: Alcohol-flavoured non-alcoholic beverage.
APVT: Adolescents’ popular viewing time.
TV: Television.
